# Supplementary material for: Seminoma and Embryonal Carcinoma Footprints Identified by Analysis of Integrated Genome-Wide Epigenetic and Expression Profiles of Germ Cell Cancer Cell Lines
Source: PLoS One. 2014 Jun 2;9(6):e98330. doi: 10.1371/journal.pone.0098330 (PMC4041891; doi:10.1371/journal.pone.0098330)
Supplement: File S1 — ZIP file containing DMRforPairs output for significant regions. Please start from the html files. (ZIP) [file pone.0098330.s008.zip › figures/21858.pdf]

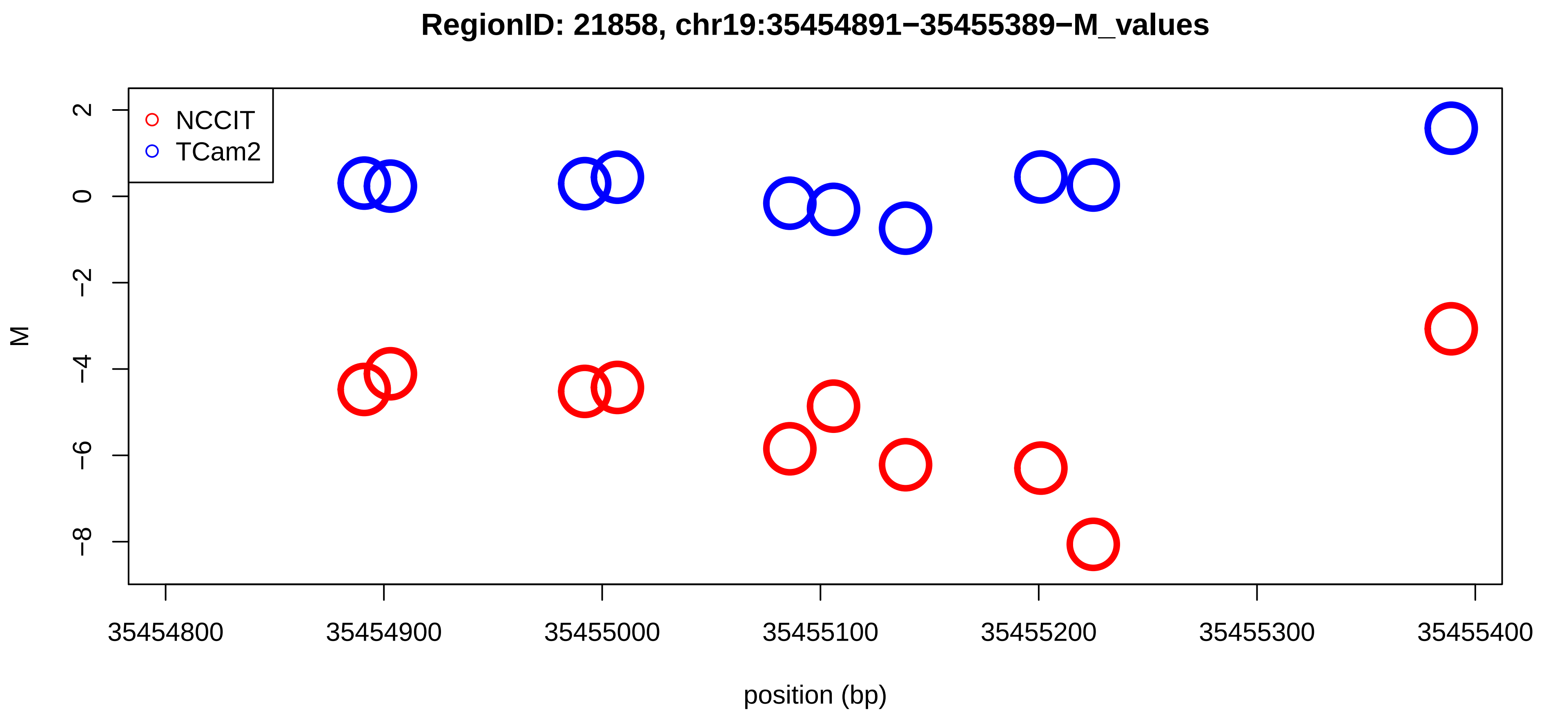
RegionID: 21858, chr19:35454891−35455389−M_values
NCCIT
TCam2
M
2
0
−2
−4
−6
−8
35454800
35454900
35455000
35455100
35455200
35455300
35455400
position (bp)

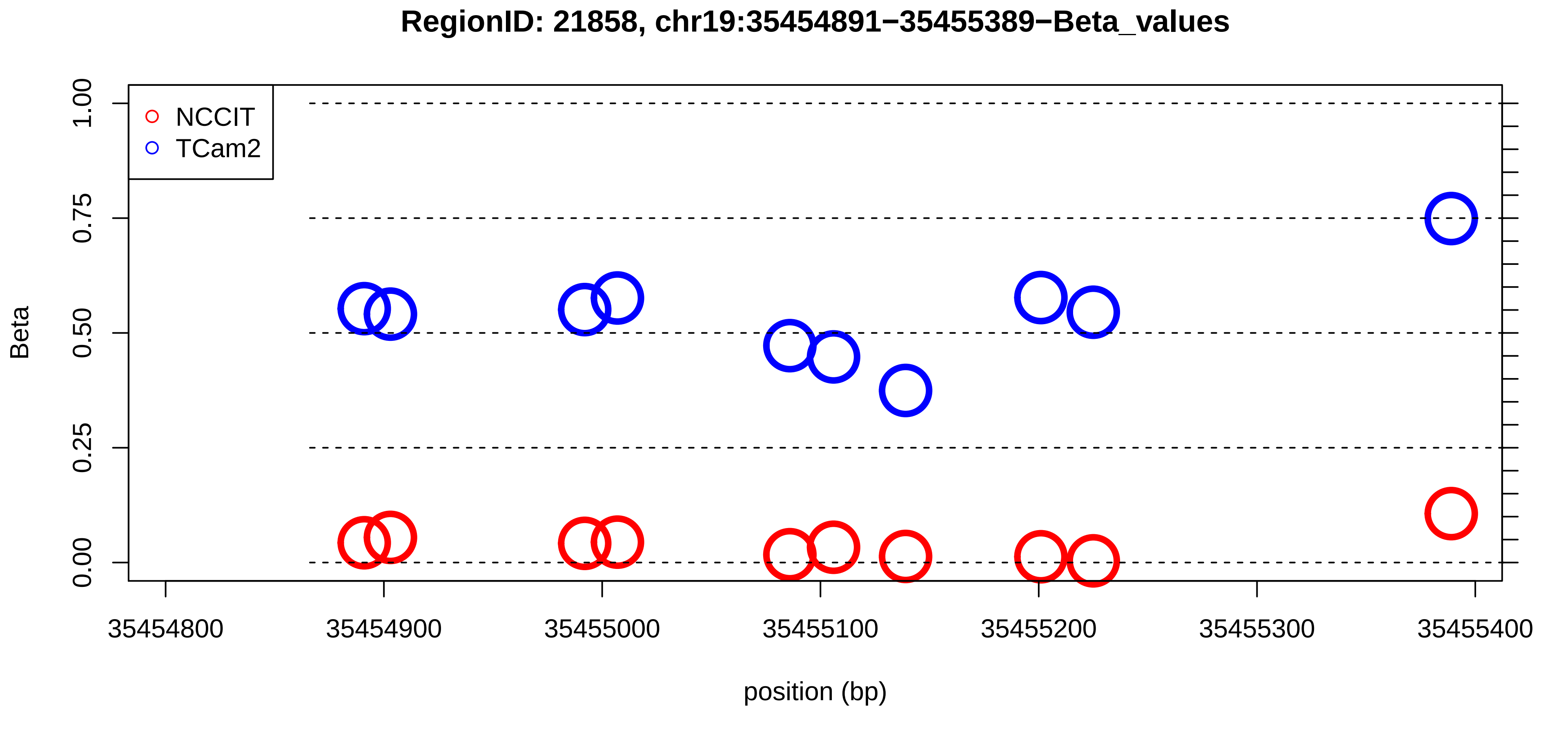
RegionID: 21858, chr19:35454891−35455389−Beta_values
NCCIT
TCam2
Beta
1.00
0.75
0.50
0.25
0.00
35454800
35454900
35455000
35455100
35455200
35455300
35455400
position (bp)
